# First record of the complete mitochondrial genome and phylogenetic analysis of *Limnodrilus hoffmeisteri* Claparède, 1862 (Annelida; Clitellata; Oligochaeta)

**DOI:** 10.1080/23802359.2021.1972485

**Published:** 2022-01-11

**Authors:** Jeounghee Lee, Jongwoo Jung

**Affiliations:** a Interdisciplinary Program of Ecocreative, Ewha Womans University, Seoul, Korea; b Department of Science Education, Ewha Womans University, Seoul, Korea

**Keywords:** Freshwater oligochaeta, *Limnodrilus hoffmeisteri*, complete mitochondrial genome, phylogenetic analysis

## Abstract

The complete mitochondrial genome of *Limnodrilus hoffmeisteri* was analyzed using the Illumina NovaSeq 6000 platform. The length of the complete mitochondrial genome was 15,972 bp. The sequencing data, comprising 13 protein coding genes (PCGs), 2 rRNA genes, 22 tRNA genes, and a putative control region, were submitted to the National Center for Biotechnology Information (MW732144). A phylogenetic tree was constructed based on the sequences of 13 PCGs using the maximum likelihood method. To date, only a single study has been conducted on the complete mitochondrial genome of another aquatic oligochaete. This phylogenetic tree revealed that *L. hoffmeisteri* is clustered with *Tubifex tubifex* and forms a sister group to the earthworm group.

*Limnodrilus hoffmeisteri* Claparède, 1862, a common species among the tubificid worms, is prevalent worldwide. Commonly found in eutrophic lakes, this species inhabits sediments, dwelling and feeding at the sediment–water interface. As the activity of *L. hoffmeisteri* has a considerable impact on the maintenance of aquatic ecosystem functions, it is considered as one of the most well-known indicators of heavy metal pollution (Ciutat et al. 2005; Kristensen et al. 2012; Chen et al. 2016). The key characteristic of *L. hoffmeisteri* is the penile sheath with head shape and length; however, it varies considerably between individuals (Ohtaka et al. 1990; Liu et al. 2017). Therefore, molecular studies have been more limited than taxonomical and environmental studies. Study of the mitochondrial genome in freshwater oligochaetes has started quite recently and is in its nascent stages. Here, the complete mitochondrial genome of *L. hoffmeisteri* was assembled and compared with other species of earthworms and annelids with known mitochondrial genome sequences. This result would provide remarkable insights into the evolutionary processes of *L. hoffmeisteri* and other aquatic oligochaete species.

The specimen was collected in Seoul (Korea) in September 2019 (127° 04′ 38.19" E 37° 34′ 53.87"N), preserved in 80% ethanol, and the voucher specimen was stored in the National Institute of Biological Resources (NIBRIV0000882544). Whole genomic DNA was extracted from posterior body segments of the adult specimen using a REPLI-g Mitochondrial DNA Kit (Qiagen, USA). The whole-genome sequencing was performed using the Illumina Novaseq 6000 platform. The mitochondrial genome was constructed using MITObim v1.9.1 (Hahn et al. 2013) and MITOS (Bernt et al. 2013). The reads were assembled and annotated using Generous Prime v. 2019.2.1 (Kearse et al. 2012), and then, all protein coding genes (PCGs) were aligned using the MAFFT algorithm (Katoh and Standley 2013). Alignment of 27 species genome data—two freshwater oligochaetes (*L. hoffmeisteri* Claparède, 1862 and *Tubifex tubifex* (Müller, 1774)), 13 earthworms (*Amynthas cucullatus* (Hong and James 2009)* A. hupeiensis* (Michaelsen, 1895), *A. jiriensis* ( Song and Paik 1971), (Qiu 1988), *A. robustus* (Perrier, 1872), *A. triastriatus* (Chen, 1946), *Drawida japonica* (Michaelsen, 1892), *Duplodicodrilus schmardae* (Horst, 1883), *Lumbricus rebellus* Hoffmeister, 1843, *Metaphire californica* (Kinberg, 1867), *M. guillelmi* (Michaelsen, 1895), *Perionyx excavates* (Perrier, 1872), and *Pontoscolex corethrurus* (Müller, 1857)), five leech species (*Erpobdella octoculata* (Linnaeus, 1758), *E. japonica* (Pawlowski, 1952), *Whitmania acranulata* (Whitman, 1886), *W. laevis* (Baird, 1869), and *Zeylanicobdella arugamensis* de Silva, 1963), five polychaetes (*Aphrodita australis* Baird, 1865, *Chaetopterus variopedatus* (Renier, 1804), *Cirriformia* cf*. tentaculata* (Montagu, 1808), *Clymenella torquata* (Leidy, 1855), and *Namalycastis abiuma* (Grube, 1872)), and two outgroup species (*Phascolosoma esculenta* (Chen & Yeh, 1958) and *Urechis caupo* Fisher & MacGinitie, 1928— was performed using MUSCLE Alignment. (Thompson et al. 2002). Maximum-likelihood (ML) analysis was conducted using PhyML 3.0 (Guindon et al. 2010); bootstrapping was 1,000 replicates in Generous Prime, with the GTR substitution model using MEGA X (Kumar et al. 2018). The *L. hoffmeisteri* mitogenome was 15,972-bp-long, and the data were submitted to NCBI (MW732144). The percentages of A, T, C, and G were 31.2%, 31.5%, 22.3%, and 15.0%, respectively. The nucleotide composition exhibited a significant bias for A + T content (62.7%). We identified 13 protein-coding genes, two ribosomal RNA genes, 22 transfer RNA genes, and a putative control region.

The phylogenetic relationship of *L. hoffmeisteri* among the Annelida was reformed (Fig. 1). The ML tree based on the *L. hoffmeisteri* mitogenome combined with previously published annelid mitogenome data revealed that *L. hoffmeisteri* is clustered with *T. tubifex* and belongs to a sister earthworm group. This result could predict the phylogenetic relationships of aquatic oligochaetes in the phylum Annelida. Overall, the relationship between major clades followed a well-published annelid phylogeny study (Achurra et al. 2011). This study presents the complete mitochondrial genome data of an aquatic oligochaete. Further studies on mitogenomes would help in substantiating the precise molecular phylogeny within the aquatic oligochaete group.

**Figure 1.**
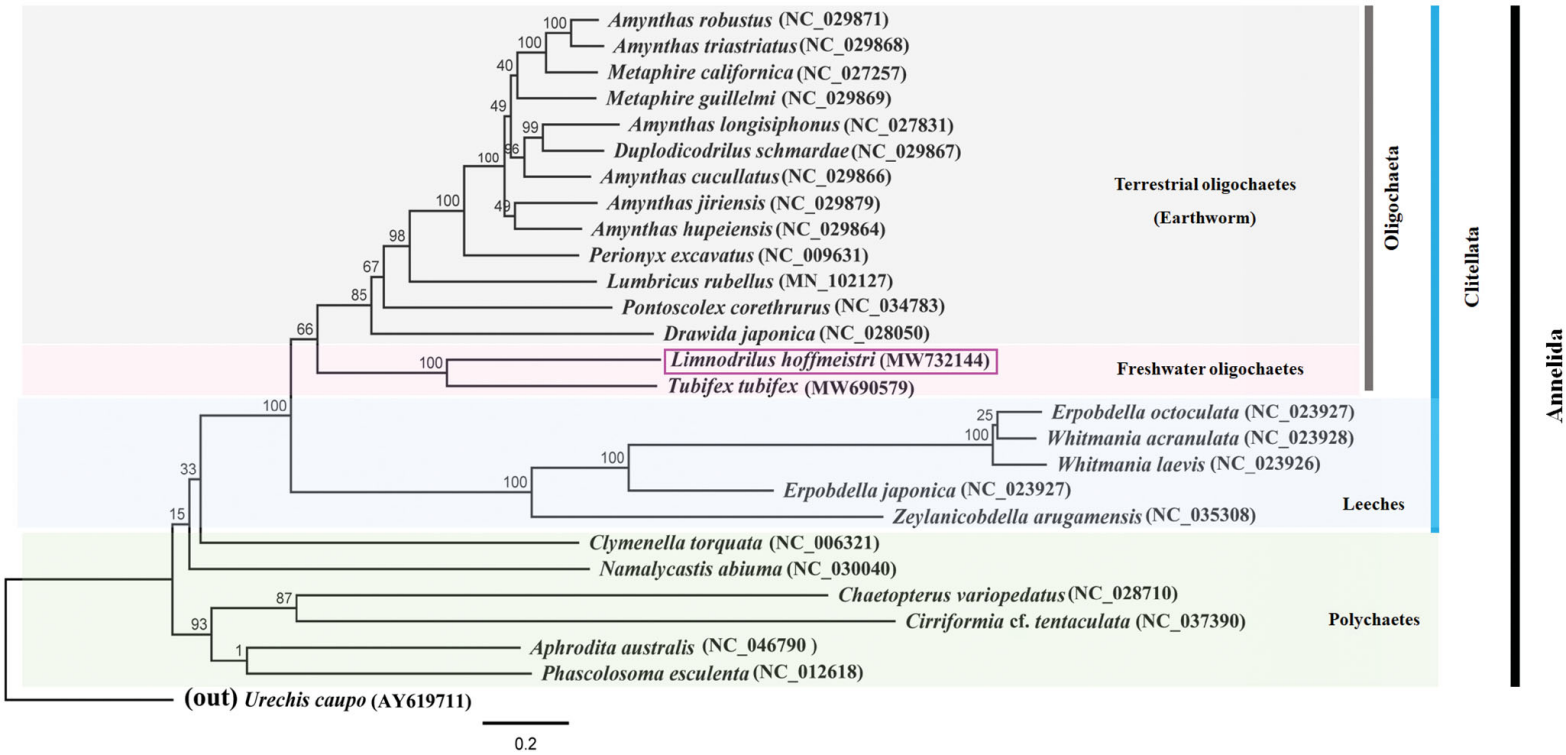
Molecular phylogeny of *L. hoffmeisteri* (MW732144), one species in freshwater oligochaetes, 13 species in terrestrial oligochaetes (Earthworm), five species in Leeches, five species in Polychaetes, and two outgroup species based on complete mitogenome. The complete mitogenomes are downloaded from GenBank and the phylogenetic tree is constructed by the Maximum-likelihood method with 1,000 bootstrap replicates.

## Data Availability

The genome sequence data that support the findings of this study are openly available in GenBank (National Center for Biotechnology Information) at https://www.ncbi.nlm.nih.gov, accession no. MW732144. The associated BioProject, SRA, and Bio Sample numbers are PRJNA726347, SRR14996617, and SAMN19953084, respectively. The data that support the findings of this study are also openly available in Mendeley Data at http://dx.doi.org/10.17632/zw7g7jnr4y.1
